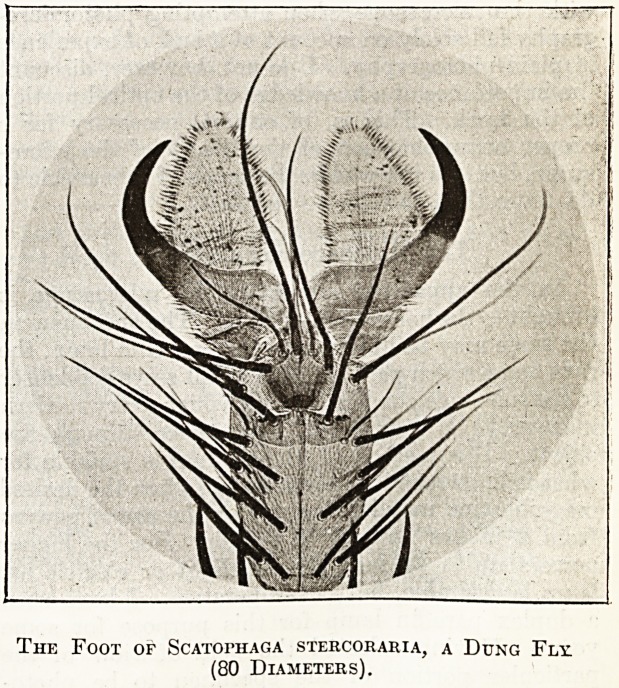# The Endless Pleasures of Photomicrography

**Published:** 1912-08-31

**Authors:** Frederick Noad Clark

**Affiliations:** Pharmacist, Paddington Infirmary.


### RELAXATIONS AND HOBBIES.

### The Endless Pleasures of Photomicrography.

By FREDERICK NOAD CLARK, Pharmacist, Paddington Infirmary.

I have always considered that photography is a pursuit eminently adapted to the calling of the pharmacist, who, by reason of his scientific training and habits of careful manipulation, is more likely to become a successful photographer than one who has not the advantage of such experience. This especially applies when the microscope is used in conjunction with photography.

The Eggs of Hadena Pisi, a Noctuid Moth (20 Diameters).

### Photography 40 Years Ago.

My experience in photography dates back to the early seventies, when my father, a professional photographer, practised the art in what is now sometimes referred to as "the good old collodion days." Extreme care and cleanliness in every detail were a *sine quâ non*; for photography in those days was very far removed from the ready-made plate and "press-the-button" system of to-day, and there are many able photographers now who extol the good points of the collodion process. It is, as a matter of fact, still largely employed in process reproduction work and in lantern-slide manufacture.

The modern gelatine dry-plate was in fairly general use about 1880, and I remember preparing the emulsion for this then new process. In 1891 I was induced by my good friend and mentor, the late Dr. T. D. Savill, who was investigating the cause of an outbreak of epidemic eczema or dermatitis in the Paddington Infirmary, to take up the subject of photomicrography. Curious to say my first essay was in the most difficult branch of work that one could start with—viz., the high-power $\frac{1}{12}$-inch oil immersion. The organism discovered was photographed at 1,000 diameters, then to me a great accomplishment, but I am inclined to think success was due more to luck than to judgment. Then followed a series of failures under comparatively simple conditions, such as the photography of histological and pathological sections. My inclinations, however, led me to take up the study of structural entomology, and in 1895 I joined the South London Entomological Society, and in my presidential year, 1902, worked out the life history of argulus, the fish-louse, illustrated by photography.

### Its Application to Natural History.

Amongst the many applications of photography I can conceive of none more interesting than that of natural history in general and entomology in particular. In the earlier days of illustrated works on the latter subject, there was no other method available but drawings and diagrams. Now, however, thanks to photography, every work of importance not only in this branch of science but in practically every other is illustrated by this means. This has been contributed to in no small degree by the perfecting of process reproduction methods. Many reproductions from drawings and diagrams are excellent in their way, but for accuracy of delineation are not to be compared with the results obtainable by photography. By its aid structure and detail are reproduced with absolute truth: detail that it would be well-nigh impossible to portray by any other means.

The Foot of Scatophaga stercoraria, a Dung Fly (80 Diameters).

Absence of the personal element is also a point that should be borne in mind.

There can, of course, be no question of the importance of a knowledge of the microscope and its use in relation to the everyday duties of the medical staff of a modern institution: whether it be regarded as an instrument of research, in the investigation of pathological conditions, in the science of bacteriology, or in the examination of urine, articles of food, and so on. As a mere hobby, too, the microscope affords unlimited interest and instruction. But it is in the application of photography to microscopy that my own hobby has more especially lain.

### Photomicrography in the Franco-Prussian War.

The term photomicrography is applied to the method by which the microscopic image is enlarged and reproduced by photography. The term microphotography is, or was, applied to the reductions of large objects by the microscope photographed on a tiny glass slip such as is fitted in fancy penholders and trinkets. The same process was utilised in the Franco-Prussian War for the conveyance of messages from Paris when under siege by means of carrier pigeons, as on account of the small bulk of the reduced photographs a large number of messages were sent in this way attached to the pigeons. The process employed was the collodion in very thin films afterwards magnified.

The formidable name photomicrography, however, need not deter anyone possessed of an average knowledge of the microscope and of practical photography from attaining fair results with a student's microscope and a simple camera. It is when photography with the higher powers of the microscope is attempted, such as for bacteria and other minute objects that the utmost skill of the worker is called for. To produce really good results the microscopist must be a practical photographer. Many experts with the microscope when attempting photomicrography fail solely on account of a lack of experience in plain photography. I do not, however, discount the importance of a knowledge of the optical portion of the work. This is, of course, necessary for a proper comprehension of the theory of the microscope, but it is not within the scope of this article to do more than to refer to the fact.

### The Microscopist at Work.

In the apparatus employed the microscope is brought to the horizontal position. The specimen on the stage may be illuminated by a paraffin lamp, the rays from which pass through a bull's-eye condenser to the sub-stage condenser, with which every modern microscope is now fitted, and thence through the object. This method of illumination is suitable for what is known as low-power work, when the desired magnification may be obtained by the use of powers from 2 inches up to $\frac{1}{2}$ or $\frac{1}{4}$ inch. For the higher powers up to $\frac{1}{12}$ inch the limelight or electric arc lamp is desirable, but not imperative. I have used a duplex paraffin lamp for this purpose for some years. Having selected the field of view of the particular portion of the specimen to be photographed, the eyepiece of the microscope is removed. At this point it is important to bear in mind that the ordinary eyepiece is not required for low- or medium-power work. It is only when using the higher powers, such as the $\frac{1}{12}$ inch, that a special eyepiece called a projection or a compensating eyepiece is necessary, and under these conditions the best skilled efforts of the worker are called for. The upper end of the draw tube of the microscope is then inserted in the camera front after removing the photographic lens, and the junction between microscope and camera is made light-tight in any way that the worker's ingenuity may suggest. The source of light, condensers, and objective must be placed centrally, so that the projected image is seen to occupy an equally illuminated and central position on the ground-glass screen of the camera. Any simple quarter-plate size camera will be suitable, if it be capable of extension, but for increased amplification the camera should be extended a considerable distance from the microscope tube. In practice it will be found that the ground-glass screen is too coarse for accurate focussing, so one substitutes for it a piece of plain glass and uses an ordinary photographic focussing lens to intercept the aerial image, and thus to obtain critical definition. A long extension of camera will necessitate the use of a focussing rod fixed to the base-board of the apparatus and connected to the fine adjustment of the microscope by a small endless band.

### Advice to a Beginner.

The beginner will be well advised to attempt at first only low-power work, for in using the higher powers difficulties of a technical character crop up which tend to discourage him. In the photography of histological and pathological sections it is desirable to have these cut as thinly as possible and mounted perfectly flat under very thin cover glasses, with the staining as "contrasty" and "discrete" as possible. Overstained and especially understained sections render photography extremely difficult. Logwood stains are very suitable and so are preparations stained by Gram's method for high-power work and bacteria.

As for the purely photographic procedure, backed chromatic plates should be used, and for faintly stained blue preparations a yellow screen interposed between the light and sub-stage is necessary. It would be difficult to give the reader any precise guide as to the length of exposure required, depending, as it does, on the character of the subject, the source of illumination (whether paraffin, limelight, or electricity), the kind of objective used, and the amount of magnification. A few trial plates will, however, soon give one a clue. In an article of this kind one has to be content with merely mentioning the special applications of photomicrography to the study of histology, zoology, geology, mineralogy, chemistry, and many other sciences where the use of the microscope is indicated.

The accompanying illustrations convey but a faint idea of the possibilities of this fascinating subject. The eggs of insects, for example, are some of the most beautiful objects in Nature, and whole volumes might be written about these alone; but the naturalist who uses the microscope as an aid to his studies will find among the endless variety of structure presented by the insect world alone an inexhaustible source of knowledge and pleasure.

## Figures and Tables

**Figure f1:**
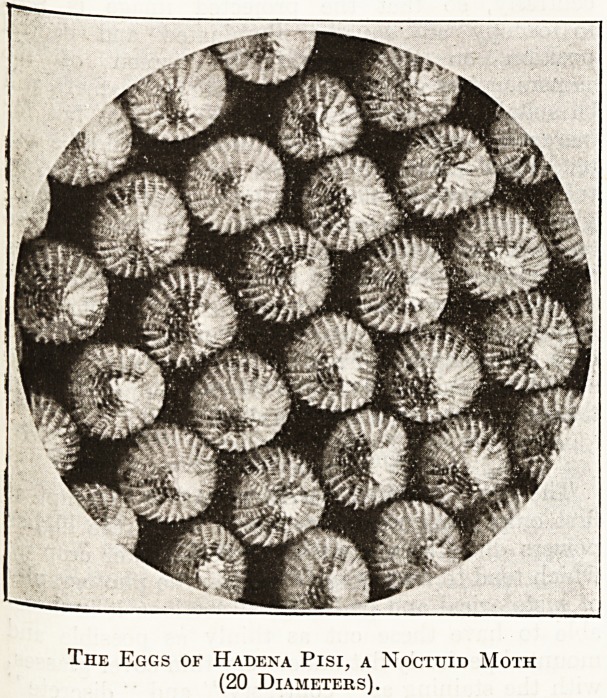


**Figure f2:**